# Cardiac dyssynchrony and response to cardiac resynchronisation therapy in heart failure: can genetic predisposition play a role?

**DOI:** 10.1007/s12471-015-0766-6

**Published:** 2015-12-08

**Authors:** N. Lahrouchi, C.R. Bezzina

**Affiliations:** Department of Experimental Cardiology, Room K2–116, Meibergdreef 9, 1105 AZ Amsterdam, The Netherlands

**Keywords:** Cardiac resynchronisation therapy, Genetics, Polymorphism

## Abstract

Cardiac resynchronisation therapy (CRT) is an accepted treatment for heart failure patients with depressed left ventricular (LV) function and dyssynchrony. However, despite better clinical outcome and improved cardiac function after CRT in the majority of eligible heart failure patients, a large proportion of implanted patients do not seem to benefit clinically from this therapy. In this review we consider whether genetic factors may play a role in modulating response to CRT and summarise the few genetic studies that have investigated the role of genetic variation in candidate genes.

## Introduction

Patients with heart failure and decreased function often develop dyssynchronous contraction of the ventricles because of electric activation delay. This further decreases systolic function and chamber efficiency and contributes to morbidity and mortality [1]. Artificial electrical stimulation to restore synchronous ventricular activation, referred to as cardiac resynchronisation therapy (CRT), which was developed in the 1990s is now an established treatment for heart failure patients with a left ventricular ejection fraction (LVEF) ≤ 35 % and a broad QRS complex (left bundle branch block) on the surface ECG [2, 3]. Yet, despite better clinical outcome and improved cardiac function after CRT in the majority of eligible heart failure patients, approximately 30 % of implanted patients do not seem to benefit clinically from this therapy (often termed non-responders) [4, 5].

## Do genetic factors play a role?

**Figure 1:**
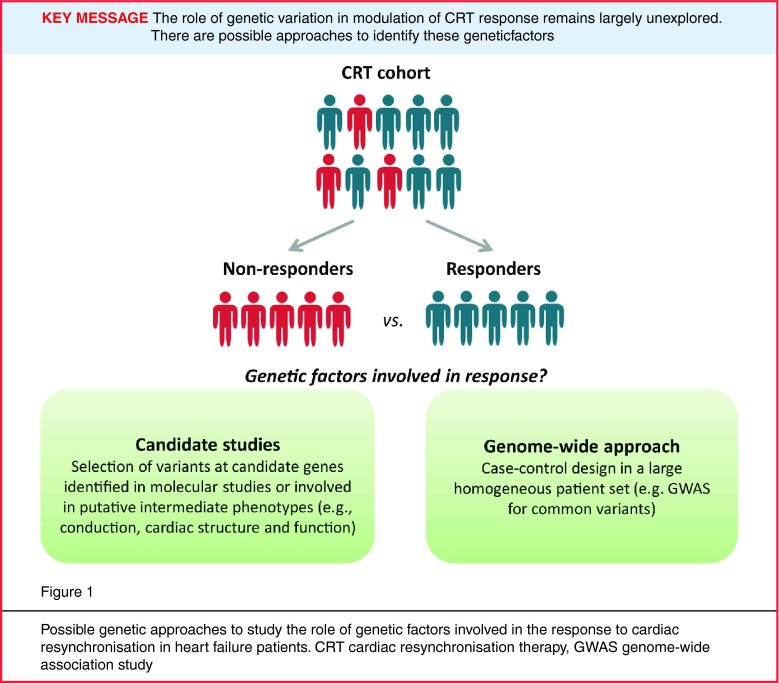

Significant interest exists in the identification of factors that predict response to CRT, as these may contribute to tailoring of the treatment of heart failure in the individual patient. Several demographic, clinical and procedural variables that could be possible predictors of CRT success have been intensively investigated. Factors implicated in response to CRT include the aetiology of heart failure, gender, QRS duration and morphology, and myocardial scar burden [2, 6]. As far as we could determine, evidence for genetic modulation of CRT treatment success, or for the development of dyssynchrony in heart failure, is non-existent; the nature of the dyssynchrony and CRT response phenotype makes heritability studies (which typically investigate clustering of the phenotype among related individuals) to demonstrate this highly challenging to conduct. However, evidence for the presence of heritable factors in the predisposition to other, related, complex cardiovascular traits, such as heart failure [7] and sudden cardiac death [8, 9], indicates that a genetic component also likely plays a role in modulating susceptibility to these phenotypes. Further support for the possible role of genetic factors stems from clear evidence of heritability of clinically measurable phenotypes that are underpinned by biological processes that likely play a role in mediating dyssynchrony or CRT response (often referred to as intermediate phenotypes). These include electrocardiographic conduction parameters, including QRS-interval duration [10], and parameters of cardiac structure and function [11]. Besides potentially contributing to the identification of patients who will benefit from CRT, the identification of the genes involved in determining response to CRT would also provide inroads for an increased understanding of the molecular pathways underlying reverse cardiac remodelling after CRT, which may eventually provide leads for the development of more effective pharmacological therapy. Yet, studies aimed at identifying genetic factors have been scant and the role of genetic variation in modulation of CRT response so far remains largely unexplored (Fig 1).

## What is the likely genetic architecture of response to CRT?

The genetic architecture of the CRT response phenotype is undoubtedly complex as demonstrated for other clinical cardiovascular traits such as heart failure [12], atrial fibrillation [13] and sudden cardiac death [14]. In addition to its complex genetic architecture it will undoubtedly be further influenced by comorbidities (such as hypertension and diabetes), medication use and environmental factors. This multifactorial aetiology and the possible interaction among different contributing factors will complicate the identification of the genetic factors involved, as genetic association studies comparing responders to non-responders may be confounded by differences in such exposures. Multiple genetic variants, both common and rare (denoting the frequency of the minor allele in the general population), are expected to contribute. Common variants, typically defined as those with a minor allele frequency > 1 %, are anticipated to contribute modestly to CRT response, whereas according to the prevailing complex inheritance paradigm, rare variants will be associated with larger effects. In terms of class of genetic variation, contributing common and rare variants may entail single nucleotide variants (the most prevalent class of genetic variation among individuals) as well as structural variants (including insertion deletions and copy number variants) [15]. Modulatory genetic variation will ultimately affect CRT response by either affecting the function of a protein (in case of genetic variation leading to alterations in the amino acid sequence of a protein) or by affecting the level of expression of a protein through effects on gene expression (in the case of genetic variation affecting regulatory regions of the genome).

## Candidate gene studies

As far as we could determine, three studies have so far searched for genetic factors modulating CRT response. All three investigated the effect of common genetic variants in candidate genes. One study focused on 7 genetic variants at 5 genes from the renin-angiotensin-aldosterone system (RAAS) [16], a pathway that plays a major role in the pathophysiology of heart failure through increased vasoconstriction, sodium and water retention, myocardial fibrosis, and ventricular remodelling [17]. The variants selected in this study had previously been associated with heart failure and left ventricular hypertrophy, amongst other traits [18]. The study, conducted in 156 patients treated with CRT (80 responders, 76 non-responders, matched by age, sex, heart failure aetiology, NYHA functional class and LVEF), defined CRT response/reverse remodelling as a > 15 % decrease in left ventricular end-systolic volume (LVESV) at 9 months after CRT. The minor allele frequency of the rs5522 common genetic variant in *NR3C2*, encoding the mineralocorticoid receptor, was found to be higher in the group that did not display reverse remodelling according to the criteria used in the study. The same case-control set used in the above study was subsequently used to test an additional 5 candidate SNPs that had been previously associated with a variety of cardiovascular disease phenotypes [19]. Of these, 3 genetic variants (rs3766031-*ATPIB1*, rs5443-*GNB3*, and rs7325635-*TNFSF11*) appeared to be associated with success of CRT. Following pre-clinical observations made in dog [20] and clinical studies [21], suggesting a role for β-adrenergic receptors in modulating response to CRT, Pezzali and co-workers [22] studied the role of 3 genetic variants at the genes encoding the β1 and β2 adrenergic receptors (*ADRB1*-Arg389Gly, *ADRB2*-Arg16Gly and *ADRB2*-Gln27Glu). They conducted their study in a consecutive cohort of 101 heart failure patients who underwent CRT or CRT-D (70 patients) implantation and who were assessed at baseline and after a follow-up of one year. They demonstrated that carriership of the Glu27 allele of the *ADRB2*-Gln27Glu polymorphism was associated with a greater increase in LVEF, and in an additional analysis was associated with less cardiac death and appropriate ICD discharge. Interestingly, the Glu27 allele has also been shown to predict a positive reverse remodelling response to the β_1_/β_2_/α_1_-blocker carvedilol in heart failure patients [23–25], and with decreased risk of sudden cardiac death [26]. It is important to stress that although the above studies are based on relevant hypotheses, the associations that emerged should be considered exploratory and highly speculative. Both CRT patient sets studied are small, which on the one hand means that they are underpowered to uncover a legitimate association of common variants with small effect and, on the other hand, may lead to false-positive findings. Furthermore, no data were presented concerning the replication of the associations in independent patient sets. The robustness of the reported associations will therefore require validation in future studies. Notwithstanding, the small effect size associated with these variants precludes their applicability in the clinical setting at this point in time.

## MicroRNAs

Two recent studies investigated the role of microRNAs (miRNAs) in CRT response [27, 28]. MicroRNAs are a family of small non-coding RNAs that have an important role in the posttranscriptional regulation of gene expression. Since miRNAs are also found to circulate in a stable form in the blood, they have the potential to serve as biomarkers for disease and monitoring of therapy [29]. In one study, miRNAs previously shown to modulate heart failure were found to be differentially expressed in the plasma of CRT responders (defined as a reduction of LVESV > 10 % and an increase of LVEF ≥ 10 %) versus non-responders [27]. A more recent study provided initial evidence that baseline miR-30d levels are associated with a favourable CRT response (defined as an increase of LVEF ≥ 10 %) [28]. Subsequently, these investigators demonstrated a cardiac origin of this miRNA and in cellular studies provided evidence for a role in cardiac biology through molecular pathways mediating hypertrophy and protective against apoptosis. Despite these promising findings, further studies are needed to establish the role of circulating miRNAs as a biomarker for predicting the success of CRT in heart failure [30].

## Pathways involved in dyssynchrony and response to CRT

Data on mechanisms underlying dyssynchrony and differential response to CRT are currently scarce and there would be a clear benefit in this regard if genetic studies were to identify possible molecular players. Ion channel or gap junction dysfunction that promotes conduction delay is clearly a highly likely candidate for the development of dyssynchrony. Similarly, structural and contractile proteins are likely involved in the development of heart failure following dyssynchrony. It is plausible that these same pathways may determine response. Gene expression studies in serial biopsies obtained pre- and post-CRT in patients have identified changes in the expression of genes encoding components of Ca^2+^ handling, *β*-adrenergic receptors, contractile proteins and myocardial natriuretic peptide [31–33]. In addition, a study conducted in dogs demonstrated that dyssynchrony causes alterations in the myocardial transcriptome that were corrected by CRT [34]. Other experimental studies conducted in animal models have uncovered a number of distinct biological processes that are reversed by CRT; these involve amongst others calcium handling, mitochondrial dysfunction, beta-adrenergic responsiveness, cell survival signalling and electrophysiological changes (reviewed in detail by Kirk and Kass, and Cho et al. [1, 35]). Electrophysiological changes are particularly interesting to understand in view of the fact that CRT not only enhances systolic function but also counteracts malignant arrhythmia [36]. Yet, further studies are needed to determine whether gene expression changes and changes in biological processes observed after CRT in these studies are truly induced by the resynchronisation therapy itself or whether they are epiphenomena of improved left ventricular function. Notwithstanding, one could hypothesise that inter-individual differences in the ability to evoke adequate molecular responses through such pathways, which may very well be genetically determined at least in part, will play a role in inter-individual variability in response to CRT.

## Identification of genetic factors predicting response to CRT: challenges and future directions

While the identification of genetic factors that modulate response to CRT is a relevant quest, since it may contribute to the ability to distinguish responders from non-responders, their identification is highly challenging considering the complexity of the phenotype and the likely complex underlying genetic architecture. In practical terms a number of obstacles, therefore, need to be overcome to enable such genetic studies. These relate primarily to the necessity of large biobanks of deeply and uniformly phenotyped patients. Once such biobanks become available, we can then dissect the possible role of genetic factors through the investigation of candidate pathways (as directed by mechanistic studies in model systems and knowledge on intermediate phenotypes), or in an unbiased genome-wide fashion using current genomic technologies (Fig. 1). Such approaches have been proven successful in studies which identified genetic factors associated with drug response across multiple disorders [37].

### Disclosures

The authors are supported by research grants from the Center for Translational Molecular Medicine (CTMM, COHFAR project) and from the Netherlands CardioVascular Research Initiative (CVON; the Dutch Heart Foundation, Dutch Federation of University Medical Centres, the Netherlands Organisation for Health Research and Development and the Royal Netherlands Academy of Sciences) (PREDICT project).

### Conflict of interests

None declared.
